# A novel recombinant ORF7-siRNA delivered by flexible nano-liposomes inhibits varicella zoster virus infection

**DOI:** 10.1186/s13578-023-01108-1

**Published:** 2023-09-12

**Authors:** Jiawei Pei, Ye Tian, Wei Ye, Jiangfan Han, Yamei Dang, Tong Cheng, Wei Wang, Yipu Zhao, Weiliang Ye, Shuyuan Huangfu, Yu Li, Fanglin Zhang, Yingfeng Lei, Airong Qian

**Affiliations:** 1 key Lab for Space Biosciences and Biotechnology, School of Life Science, Northwestern Polytechnical University, Xi’an, Shaanxi China; 2 Department of Microbiology, School of Preclinical Medicine, Airforce Medical University: Fourth Military Medical University, Xi’an, Shaanxi China; 3 State Key Laboratory of Molecular Vaccinology and Molecular Diagnostics, National Institute of Diagnostics and Vaccine Development in Infectious Diseases, School of Life Sciences, School of Public Health, Xiamen University, Xiamen, China; 4 Department of Pharmaceutics, School of Pharmacy, Fourth Military Medical University, Xi’an, Shaanxi China; 5 Institute of Medical Research, Northwestern Polytechnical University, Xi’an, Shaanxi China

**Keywords:** Varicella zoster virus (VZV), Recombinant siRNA, r/si-ORF7, Flexible nano-liposomes, 3D human epidermal skin model

## Abstract

**Background:**

Varicella zoster virus (VZV), which is a human restricted alpha-herpesvirus, causes varicella (chickenpox) and zoster (shingles). The subsequent post-herpetic neuralgia (PHN) due to VZV infection is excruciating for most patients. Thus, developing specific therapeutics against VZV infection is imperative. RNA interference (RNAi) represents an effective approach for alternative antiviral therapy. This study aimed to develop a novel anti-VZV therapeutics based on RNAi.

**Results:**

In this study, we screened and found the open reading frame 7 (ORF7) of the VZV genome was an ideal antiviral target based on RNAi. Therefore, a novel siRNA targeting ORF7 (si-ORF7) was designed to explore the potential of RNAi antiviral treatment strategy toward VZV. We used a bio-engineering approach to manufacture recombinant siRNA agents with high yield in *E. coli*. Then, the efficacy of recombinant ORF7-siRNA (r/si-ORF7) in inhibiting VZV infection both in cellular level and 3D human epidermal skin model was evaluated. The r/si-ORF7 was proved to inhibit the VZV replication and reduce the virus copy numbers significantly in vitro. Furthermore, flexible nano-liposomes were established to deliver r/si-ORF7 to 3D human epidermal skin model and found r/si-ORF7 also could inhibit the VZV infection, thus maintaining normal skin morphology.

**Conclusions:**

Taken together, our results highlighted that transdermal administration of antiviral r/si-ORF7 was a promising therapeutic strategy for functional cure of VZV infection.

**Supplementary Information:**

The online version contains supplementary material available at 10.1186/s13578-023-01108-1.

## Background

Varicella zoster virus (VZV), which has a linear double-stranded DNA genome with 125 kb in length, is the smallest member of all known human alpha-herpesvirus family. Its genome is comprised of at least 71 open reading frames (ORFs). Primary VZV infection causes varicella which is a highly contagious rash illness, in young children frequently, and establishes a long latency in sensory nerve ganglia thus exerting a considerable burden worldwide [1]. Upon declining immunity, VZV reactivates from latency to cause zoster with severe pain and PHN syndrome which can last for a month or even longer thus severely impacting life quality of patients [2, 3]. Although VZV infection can be controlled by antiviral medications, no effective therapy has been licensed to treat complications of VZV neuronal infection, particularly PHN. Thus, more effective, and specific antiviral drugs need to be developed urgently to provide better protection for host’s physiological functions.

RNA interference (RNAi) is a critical mechanism for organisms to defend against foreign pathogens [4–6]. Small interference RNAs (siRNAs), one of the major executors of RNAi, can specifically downregulate corresponding target mRNAs, including a product of viral infections. The specific siRNAs may be designed towards key factors of the viral replication cycle thus preventing the replication and spreading of the viruses. The rapid growth of RNAi-based clinical trials for viral infections has been witnessed in the past several years, for instance, Epstein–Barr virus (EBV) [7], hepatitis B virus (HBV) [8, 9], human immunodeficiency virus (HIV) [10], SARS-CoV [11], and the raging SARS-Cov-2 [12–14]. Hence, siRNAs can serve as powerful weapons in the fight against viral infection.

Recently, a novel hybrid tRNA^Ser^ scaffold expressing siRNAs by microbial fermentation was established, which represents a promising approach to manufacture natural siRNA molecules with high yield for siRNA therapeutics developing in a cost-effective manner [15–18]. All recombinant siRNAs were expressed at a high level in *Escherichia coli* (*E. coli*) HST08 strain and purified to a high degree homogeneity by fast protein liquid chromatography (FPLC). Distinguished from chemically-synthesized siRNA carrying extensive and various forms of chemical modifications, bioengineered siRNAs are produced and folded within living cells and comprised of a minimal level of posttranscriptional modifications that should better capture the structures, biological functions, and safety profiles of cellular RNAs. Previous studies have proved the concept that recombinant siRNAs are effective in the controlling of target gene expression in vitro and in vivo [19–26].

VZV infection causes skin lesion. Reducing the amounts of viral particles on the skin surface may alleviate pain in patients. Although, siRNA therapeutics show promise to cure skin lesion-induced VZV infection, the outer layer of the skin, the stratum corneum, represents the most resistible barrier to nucleic acids penetration across the skin, which significantly limits the absorption of siRNAs, and thus hampers the efficient delivery of siRNA payloads. Recent advancements demonstrate the power of flexible nano-liposomes for the delivery of small molecule drugs, which penetrate deeper epidermis layers through lipid lamellar areas of stratum corneum [27].

In our study, we aimed to develop a novel anti-VZV therapeutics based on RNAi.

We screened and found ORF7 was an ideal target among VZV genome for inhibiting virus replication. Next, we produced recombinant ORF7-siRNA (r/si-ORF7) using *E. coli* system and established a 3D human epidermal skin model to mimic the specific infection of VZV in human. The flexible nano-liposomes were prepared to delivery r/si-ORF7 to 3D human epidermal skin model. We demonstrated that r/si-ORF7 could inhibit VZV infection effectively. This study indicates that antiviral therapy based on RNAi has great potential for VZV treatment.

## Results

### Screening of highly specific and potent siRNAs against VZV

**Fig. 1:**
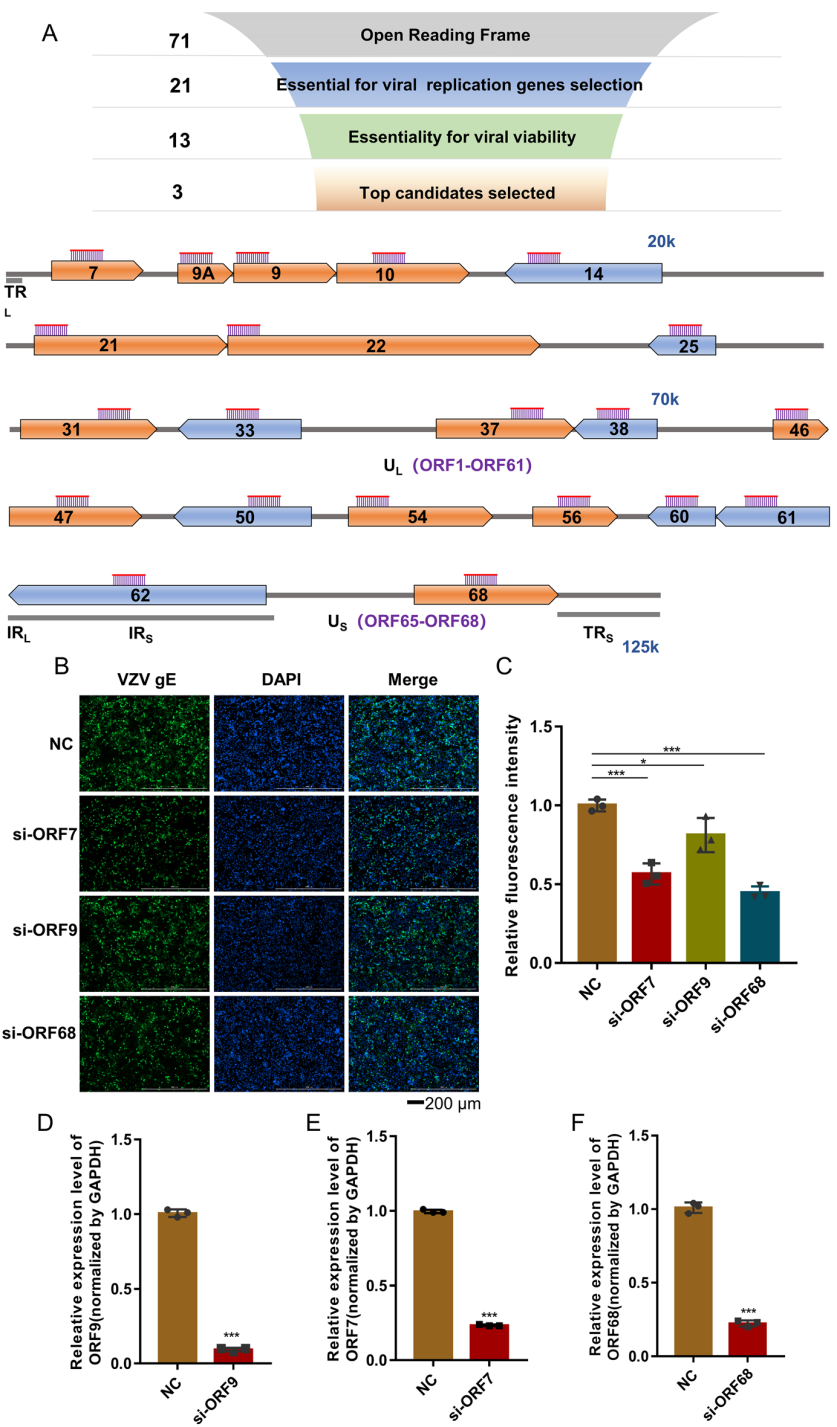
Screening of highly potent siRNAs against VZV. **A** Scheme of screening strategy. The selection criteria and sequence numbers remaining at the ending of each stage were individually indicated. The lower part is the siRNA location in the VZV genome. **B** Representative fluorescent images of VZV gE expression in ARPE-19 cells that were transfected with 40 nM of siRNAs 6 h before infection by VZV v-Oka at moi of 0.3 and incubated for 2 days. The same dose of the negative control RNA (NC) was also applied. Representative siRNAs were abbreviated as si-ORF7, si-ORF9, and si-ORF68. gE was stained green and nuclei were labeled with DAPI (blue), scale bars = 200 μm. **C** The relative fluorescence intensity in **B**. Values are the mean ± S.D. of triplicated treatments; *P < 0.05; ***P < 0.001. **D**–**F** The inhibitory capability to target gene of siRNAs. ARPE-19 cells were transfected with 40 nM of siRNA 6 h before infection by VZV v-Oka at moi of 0.3 and incubated for 2 days. The expression level of target gene was detected by RT-qPCR; ***P < 0.001

To select a highly potent and specific siRNA against VZV, a systematic strategy was applied (Fig. 1A, upper). The 21 out of 71 ORFs of VZV were considered to be essential for viral replication according to literature analysis [28]. Next, 21 siRNA candidates targeting to these ORFs of VZV Oka vaccine strain (VZV v-Oka) genomes (AB097932.1) were selected and synthesized chemically, including those coding envelope glycoprotein, tegument, DNA packaging, nucleocapsid, serine-threonine kinase, transcription regulators (Fig. 1A, lower). The effectiveness of selected siRNA to protect ARPE-19 cell against VZV infection was verified. The data showed that 13 out of 21 siRNA candidates could decrease VZV levels as indicated by glycoprotein E (gE) expression (Additional file 1: Figure S1, marked by blue), which was a late expression protein responsible for virus replication and intercellular spread [29]. Furthermore, we found si-ORF7, si-ORF9 and si-ORF68 had the strongest inhibiting capability to viral replication at a concentration of 40 nM among these 21 siRNAs (*P < 0.05, ***P < 0.001; Fig. 1B, C, Additional file 1: Figures S1, 2). The mRNA expression of ORF7, ORF9 and ORF68 decreased ~ 70% (***P < 0.001; Fig. 1D–F). Because ORF7 is a critical tropic factor required both for skin and neuronal infection of VZV, interference of ORF7 by siRNA displayed potent antiviral activity, so we selected si-ORF7 for further study.

**Fig. 2:**
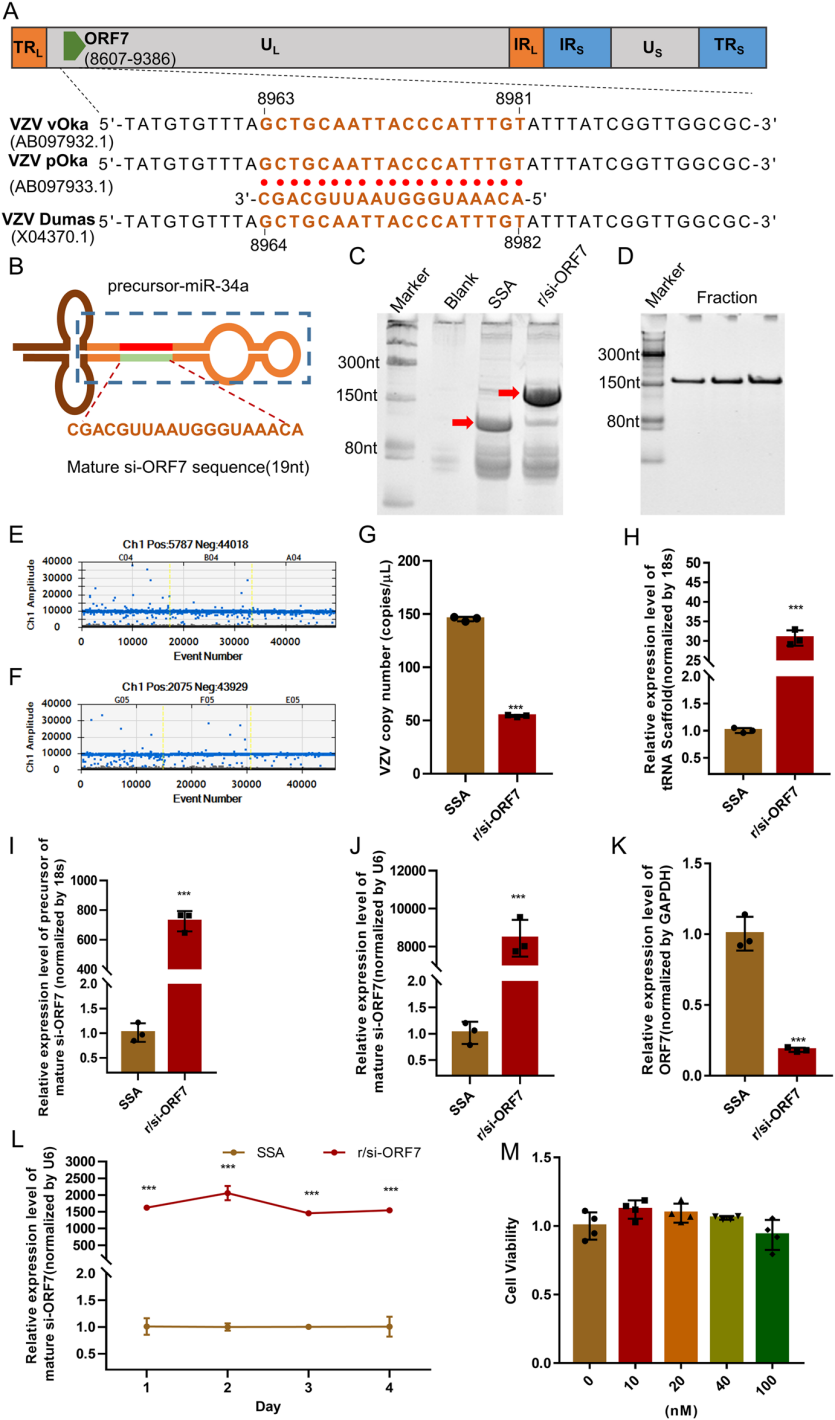
Bioengineered r/si-ORF7 was processed to mature si-ORF7 and reduced virus copy numbers. **A** A genome map for three different VZV strains. TR_L_, terminal repeat long; IR_L_, internal repeat long; U_L_, unique long, IRs, terminal repeat short; Us, unique short. A highly conserved region of the VZV ORF7 was target by si-ORF7. The target site and sequence for si-ORF7 recognition on ORF7 gene was shown below the map (si-ORF7 sequence is shown in red and the viral sequence in black). **B** Schematic illustration of the novel hybrid tRNA^Ser^ scaffold used to produce r/si-ORF7. The location of mature si-ORF7 sequence was shown in red. **C** Urea-PAGE analysis of total bacterial RNAs showed that chimeric r/si-ORF7 was heterogeneously expressed in *E. coli* using a novel hybrid tRNA^Ser^ scaffold. Total RNAs isolated from untransformed HST08 (blank) *E. coli* were used as control. r/si-ORF7 and SSA were expressed at much higher levels marked by red arrow. **D** Urea-PAGE analyses of the collected fractions eluted at 24.5 min, which confirmed the purity of isolated r/si-ORF7. **E**–**G** Virus copy numbers were detected by dPCR. ARPE-19 cells were transfected with 10 nM of r/si-ORF7 6 h before infection by VZV v-Oka at moi of 0.3 and incubated for 2 days. Positive droplets were showed in both r/si-ORF7 (**E**) and SSA (**F**) 2 days post-infection by QutantaSofts’ddPCR fluorescence readouts. **G** the copy numbers of VZV vOka (ORF68 was selected to detect). Values are the mean ± S.D. of triplicated treatments; ***P < 0.001 relative to SSA. **H**–**K** The process of r/si-ORF7 to mature si-ORF7. ARPE-19 cells were transfected with 10 nM of r/si-ORF7 6 h before infection by VZV v-Oka at moi of 0.3 and incubated for 2 days. The level of tRNA scaffold (**H**), precursor of mature si-ORF7 (**I**), mature si-ORF7 (**J**), and ORF7 mRNA expression level (**K**) were detected 48 h post-transfection. Values are the mean ± S.D. of triplicated treatments; ***P < 0.001 vs. SSA. **I** ARPE-19 cells were transfected with 10 nM of r/si-ORF7. Mature si-ORF7 expression was detected by stem loop-RT qPCR at 1, 2, 3, and 4 days post-transfection. Values are the mean ± S.D. of triplicated treatments; ***P < 0.001 vs. SSA. **M** ARPE-19 cells were transfected with 10 nM to 100 nM r/si-ORF7. CCK8 assay was undertaken to measure the cell viability of r/si-ORF7 on ARPE-19 cells

### The process of r/si-ORF7 to mature si-ORF7 and the reduction of virus copy numbers by r/si-ORF7

Based on the gene sequence posted on National Center of Biotechnology Information website, si-ORF7 was designed to complement a highly conserved region with no mutations among VZV v-Oka, VZV parental Oka (p-Oka) and VZV Dumas (Fig. 2A, upper: the location of ORF7 in the VZV genome map; lower: sequence alignment of si-ORF7 and ORF7). Subsequently, a novel hybrid tRNA^Ser^ scaffold was employed to produce recombinant ORF7-siRNA (r/si-ORF7, Fig. 2B and Additional file 1: Figure S3) in *E. coli* system [19, 24], which was a cost-effective way to obtain siRNA in large scale. To assess the expression of r/si-ORF7 in bacteria, total RNAs were extracted from HST08 *E. coli* after 16 h post-transformation with expression plasmid, and then subjected to RNA electrophoresis. As shown in Fig. 2C, a 150 nt band in r/si-ORF7 group and a smaller band in SSA (tRNA^Ser^ fused Sephadex aptamer, as negative control) group appeared, indicating a successful expression of target recombinant RNAs. After purifying by anion-exchange FPLC (Additional file 1: Figure S4), the purity of isolated r/si-ORF7 was confirmed by urea-PAGE analysis and showed a high degree of homogeneity (> 95%) (Fig. 2D).

QuantaSofts’ ddPCR was employed to accurately quantify virus copy numbers. As shown in Figs. 2E, F and 10 nM r/si-ORF7 could reduce positive droplet population above the threshold level compared to the same dose of negative control SSA. Also, copy numbers of VZV v-Oka decreased approximately 70% than SSA when r/si-ORF7 was applied (***P < 0.001; Fig. 2G). To determine whether mature si-ORF7 could be produced from r/si-ORF7 in ARPE-19 cell, selective RT-qPCR and stem loop RT-qPCR assays were conducted to determine the levels of tRNA^ser^ scaffold, precursor of mature si-ORF7, and mature si-ORF7 after 48 h transfection. The tRNA^ser^ scaffold level was significantly increased after transfection (***P < 0.001; Fig. 2H), and both the precursor and mature si-ORF7 dramatically raised as high as 600 folds (***P < 0.001; Fig. 2I), and 8000-folds (***P < 0.001; Fig. 2J) respectively compared to SSA, indicating a successful transfection and processing of r/si-ORF7. We further evaluated the biological activity of r/si-ORF7 on VZV v-Oka. The results showed r/si-ORF7 could reduce ORF7 mRNA expression to approximately 70% (***P < 0.001, vs. SSA; Fig. 2K). Interestingly, the high level of mature si-ORF7 persisted 4 days post-transfection (***P < 0.001; Fig. 2L). What’s more, the r/si-ORF7 displayed negligible cytotoxicity to ARPE-19 cells till 100 nM that was 10 folds higher than its working concentration (10 nM) used in antiviral experiments (Fig. 2M). Thus, r/si-ORF7 significantly inhibited VZV v-Oka copy numbers by silencing ORF7 gene.

### The inhibition of VZV v-Oka and r-Oka replication by r/si-ORF7 in vitro

**Fig. 3:**
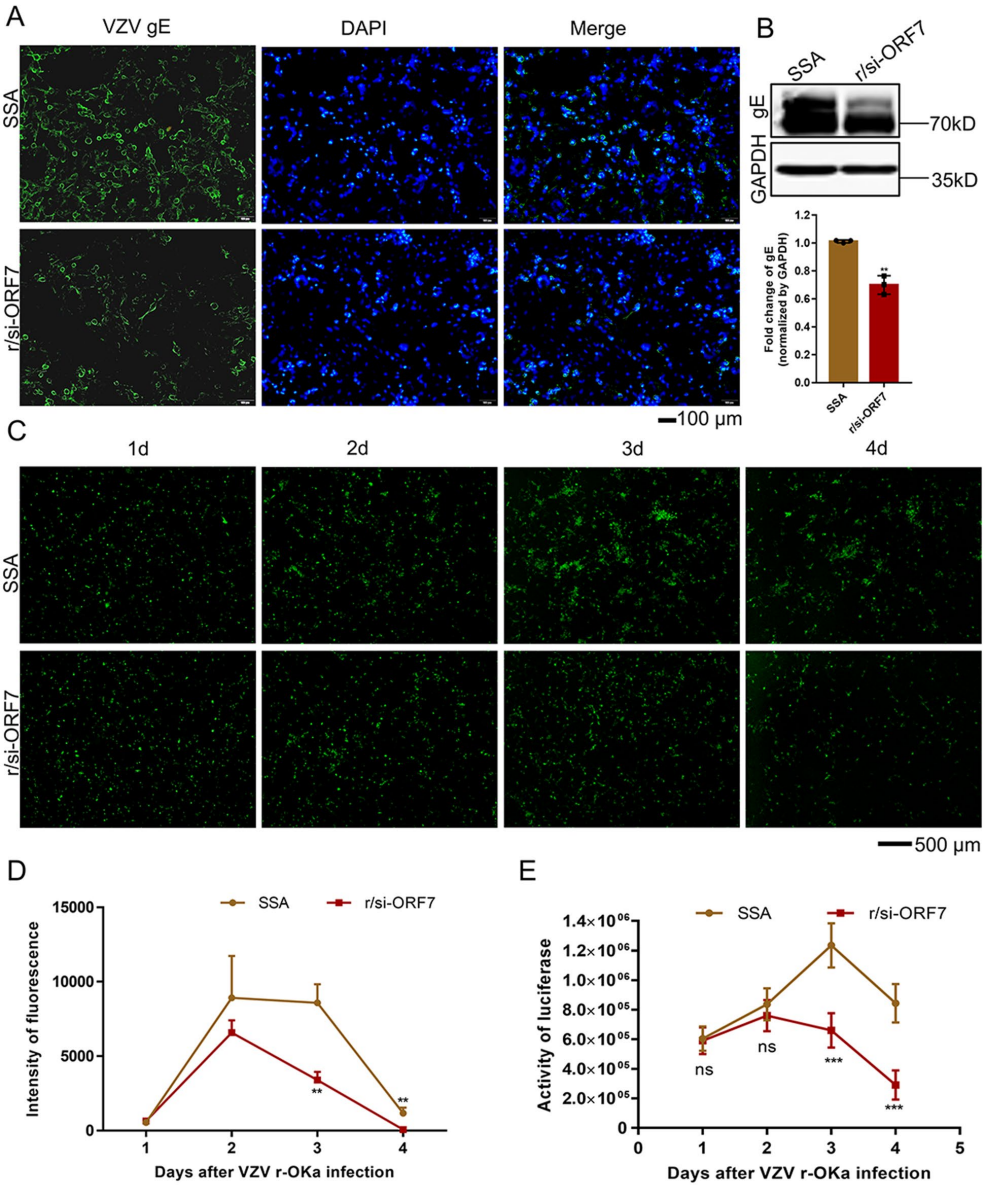
R/si-ORF7 inhibited VZV replication in human ARPE-19 cells without toxicity. ARPE-19 cells were transfected with 10 nM of r/si-ORF7 6 h before infection by VZV v-Oka (**A**, **B**) or r-Oka (**C**–**F**) at moi of 0.3 and incubated for 2 days. **A** VZV gE expression was evaluated using an immunofluorescence or **B** western blotting. gE was stained green and nuclei were labeled with DAPI (blue). Scale bar = 100 μm. **C** GFP expression was observed using a fluorescence microscope at 1, 2, 3, and 4 dpi. Scale bar = 500 μm. **D** The fluorescence intensity in **C**. Values are the mean ± S.D. of triplicated treatments; **P < 0.01 relative to SSA. **E** Activity of luciferase was detected at 1, 2, 3, and 4 dpi. ***P < 0.001 relative to SSA.

VZV v-Oka and VZV recombinant Oka (r-Oka) strains were selected to further verify the inhibition of r/si-ORF7 to viral replication. Immunofluorescence (IF) and Western blot assays (gE was selected as a marker to indicate intracellular virus level) both showed that 10 nM r/si-ORF7 could reduce VZV v-Oka level in ARPE-19 cell than the same dose of control significantly (**P < 0.01; Fig. 3A, B). To further assess antiviral activities of r/si-ORF7 on r-Oka strains (a GFP and luciferase double labeled virus), the GFP expression and activity of luciferase was monitored from 1 to 4 days post-infection (d.p.i). The data showed r/si-ORF7 could suppress r-Oka strain replication and cell to cell spread (Fig. 3C). The fluorescent clusters were significantly less in r/si-ORF7 transfected cells than that of SSA, which was more obvious on 3 and 4 d.p.i (**P < 0.01, Fig. 3C, D). The consistent tendency was also observed in luciferase activity determination (***P < 0.001; Fig. 3E). Therefore, the r/si-ORF7 reduced intracellular VZV level effectively.

### Preparation and characterization of flexible nano-liposomes

**Fig. 4:**
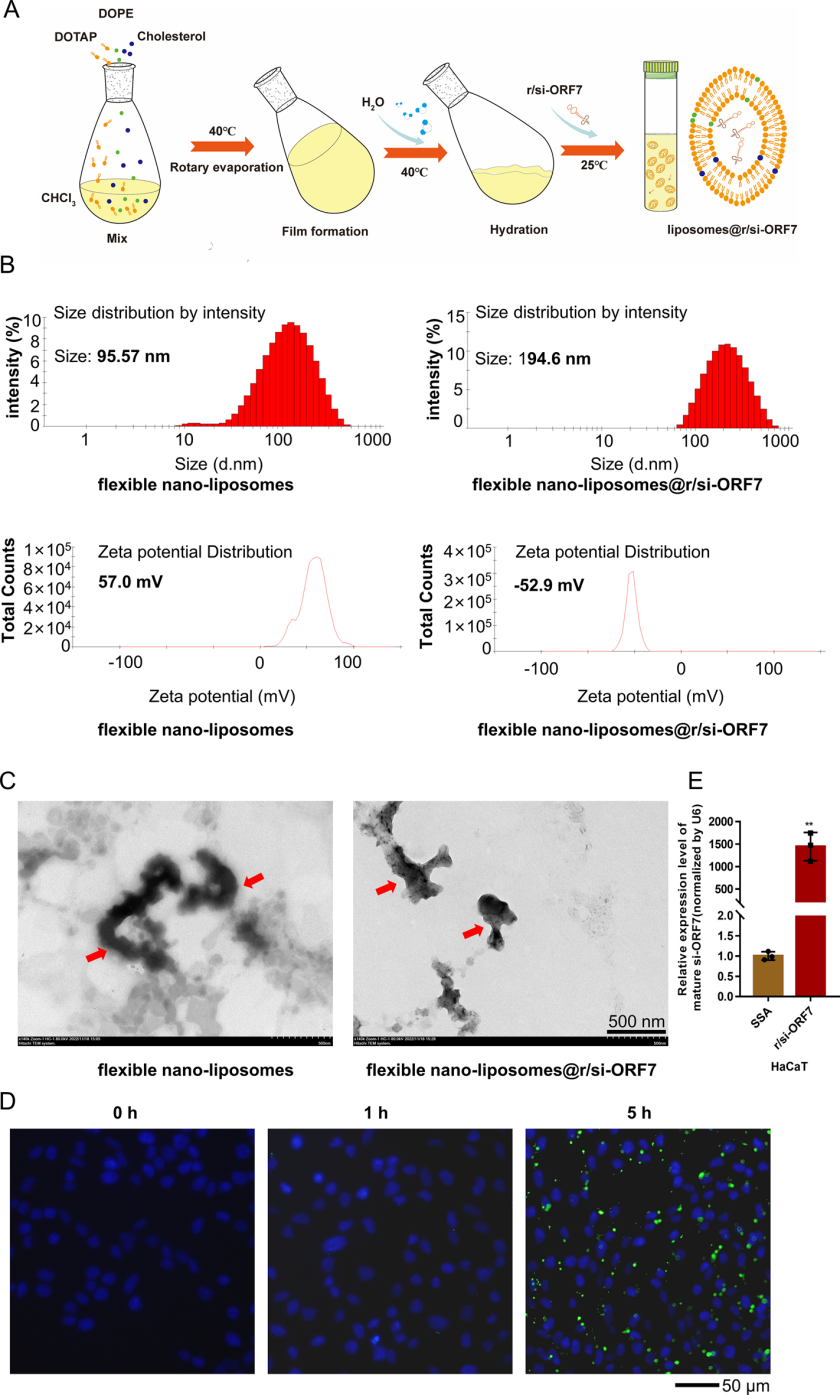
Preparation and characterization of flexible nano-liposomes. **A** Scheme of flexible nano-liposomes’ preparation. **B** Particle size distribution and zeta potential of flexible nano-liposomes and flexible nano-liposomes@ r/si-ORF7. **C** TEM image of flexible nano-liposomes (left) and flexible nano-liposomes@ r/si-ORF7 (right) complexes marked by red arrow. Scale bar = 500 nm. **D** HaCaT cellular uptake of RNA^FAM^-delivered by flexible nano-liposomes after transfection at 0, 1, 5 h as observed under fluorescence microscope (Scale bar = 50 μm). RNA^FAM^ was labled green and nuclei were labeled with Hoechst (blue). **E** The delivery efficiency of r/si-ORF7 with flexible nano-liposomes. The expression level of mature si-ORF7 was detected by RT-qPCR 48 h post-transfection

To achieve the transdermal delivery of r/si-ORF7, flexible nano-liposomes was adopted [30]. The preparation procedures of this liposomes were shown in Fig. 4A. Compared to spherical nanoparticles, fusiform nanoparticles are more conducive to the penetration of skin tissue, hence enhancing the efficiency of drug transdermal delivery. The size of flexible nano-liposomes with/without loading r/si-ORF7 were 95.57 and 194.6 nm respectively (Fig. 4B upper). And, the zeta potential of the nano-liposomes was 57.0 mV and − 52.9 mV before and after combining r/si-ORF7, respectively (Fig. 4B lower). Transmission electron microscope (TEM) analyses revealed that the flexible nano-liposomes with/without loading r/si-ORF7 showed irregular shape (Fig. 4C). Compared to spherical nanoparticles, flexible nano-liposomes are more conducive to the penetration of skin tissue owing to their superior deformability, thereby enhancing the efficiency of drug transdermal delivery. We then detected the HaCaT cell’s uptake of flexible nano-liposomes carried RNA that was tagged by FAM (5-Carboxyfluorescein, a florescent dye) at different time points. The florescent imaging revealed a significant increase of intracellular florescent intensity in 5 h (Fig. 4D), the stem-loop RT-qPCR also indicated a dramatic raise of mature si-ORF7 level after 48 h transfection (**P < 0.01; Fig. 4E), which confirmed that flexible nano-liposomes could delivery r/si-ORF7 successfully into the cells. The cytotoxicity to ARPE-19 cells of flexible nano-liposomes also were not observed by CCK8 assay (Additional file 1: Figure S5).

### The activity of r/si-ORF7 against VZV in 3D human epidermal skin model

**Fig. 5:**
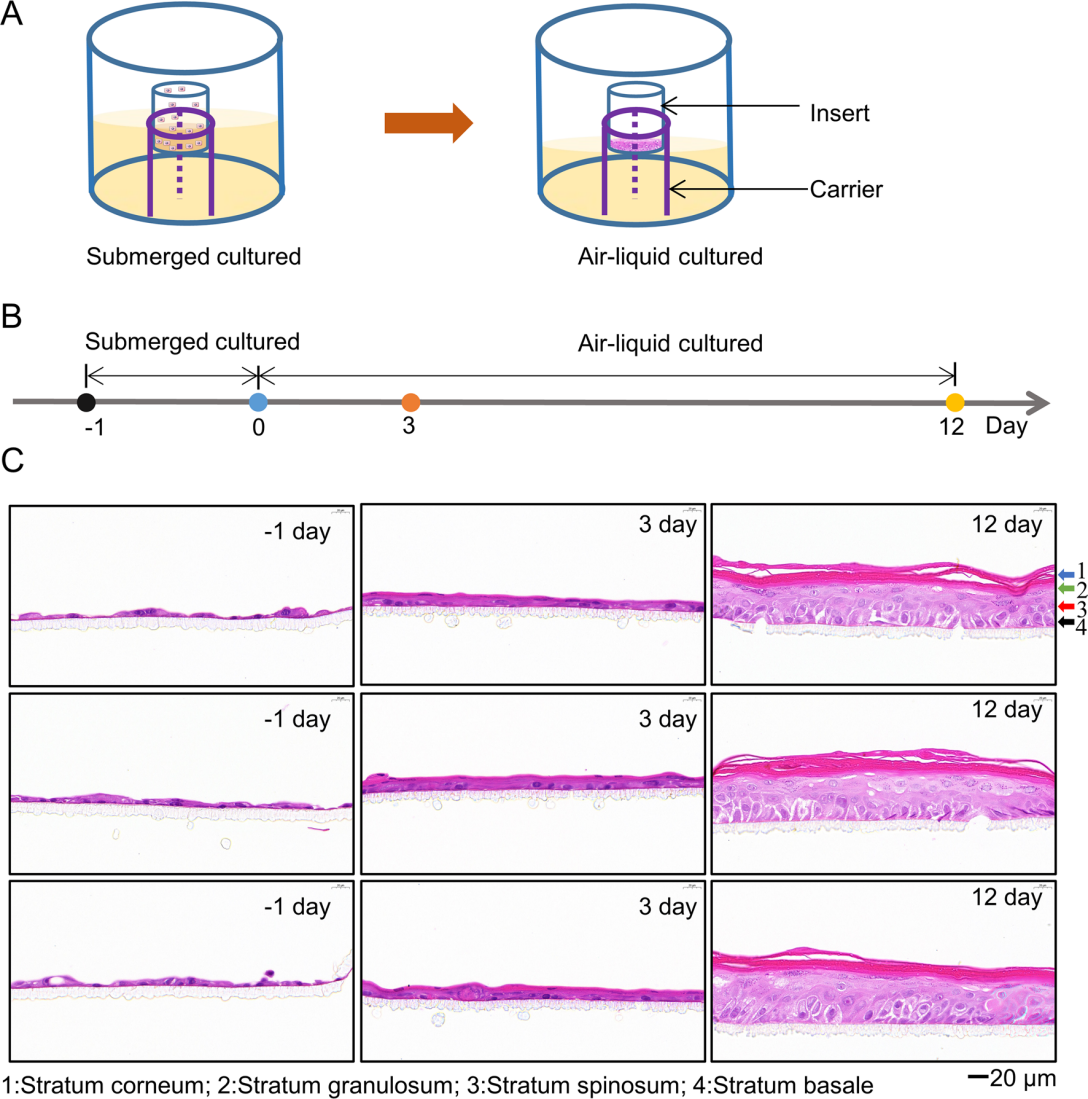
Establishment of 3D human epidermal skin model on cell culture insert in carrier system. **A** Scheme of the air-liquid interface culturing model. **B** Flowchart for establishing 3D human epidermal skin model. **C** Examination of fixed and sectioned artificial skin tissue by H&E. Stratum corneum, stratum granulosum, stratum spinosum, and stratum basale were marked by 1, 2, 3, 4 respectively. Scale bar = 20 μm

**Fig. 6:**
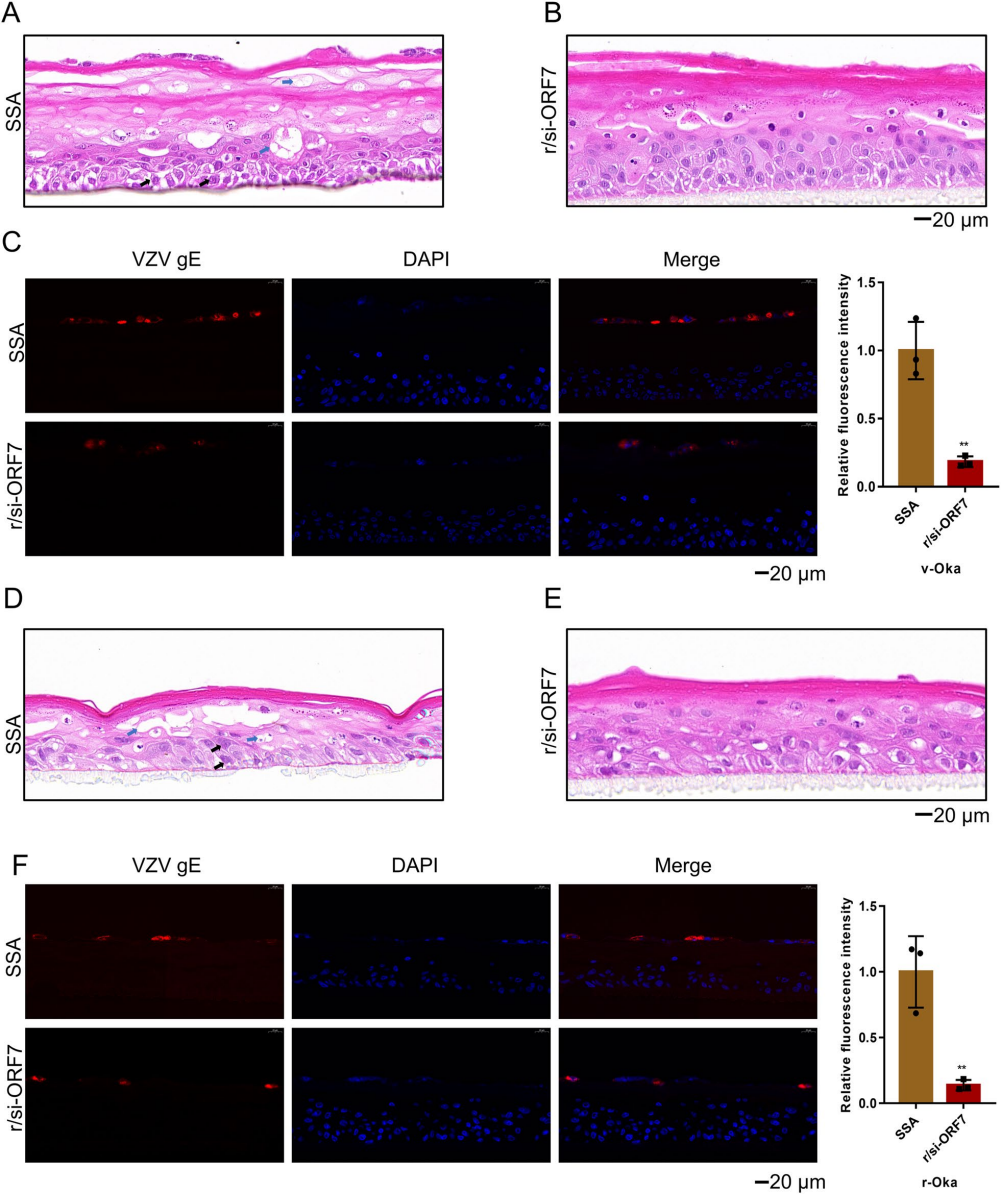
Activity of r/si-ORF7 against VZV in 3D human epidermal skin model. **A**–**C** 3D human epidermal skin model was transfected with 10 nM of SSA (**A**) or r/si-ORF7 (**B**) delivered by flexible nano-liposomes 6 h before infection by VZV v-Oka at moi of 0.3 and incubated for 2 days. Examination of fixed and sectioned artificial skin tissue by H&E, and VZV gE expression was observed using a fluorescence microscope (**C**). gE was stained red and nuclei were labeled with DAPI (blue). Vacuolation and syncytium were marked by blue and black arrow respectively. Values are the mean ± S.D. of triplicated treatments; **P < 0.01 vs. SSA; Scale bar = 20 μm. **D**–**F** 3D human epidermal skin model was transfected with 10 nM of SSA (**D**) or r/si-ORF7 (**E**) delivered by flexible nano-liposomes 6 h before infection by VZV v-Oka at moi of 0.3 and incubated for 2 days. Examination of fixed and sectioned artificial skin tissue by H&E, and VZV gE expression was observed using a fluorescence microscope (**F**). gE was stained red and nuclei were labeled with DAPI (blue). Vacuolation and syncytium were marked by blue and black arrow respectively. Values are the mean ± S.D. of triplicated treatments; **P < 0.01 vs. SSA; Scale bar = 20 μm

Since VZV is restricted to replication in human, we developed a biological model system to study antiviral activity of r/si-ORF7 (Fig. 5A, B). The examination of fixed and sectioned artificial skin tissue by microscope indicated good differentiation of the epidermal layers after submerged culture and air-liquid interface culture. All the expected cell types (stratum corneum; stratum granulosum; stratum spinosum; stratum basale) were visible in the sectioned and stained tissues (Fig. 5C), suggesting 3D human epidermal skin model was established successfully. Then, r/si-ORF7 was delivered by the flexible nano-liposomes to 3D human epidermal skin model. The activity of r/si-ORF7 against both VZV v-Oka (Fig. 6A–C) and r-Oka (Fig. 6D–F) was detected. As shown in Fig. 6A, B, r/si-ORF7 could prevent the lesions of epidermal, which showed that vacuolation increase lead to thickening of the stratum corneum and the appearance of syncytia in the basal layer (marked by blue and black arrow respectively) treated with SSA as indicating by H&E staining. IF assay also showed the consistent result, the florescence of gE was reduced greatly (**P < 0.01; Fig. 6C). Similarly, r/si-ORF7 could also inhibit the VZV r-Oka infection and protect epidermal skin structure from VZV r-Oka damage (**P < 0.01; Fig. 6D–F). Thus, r/si-ORF7 had potent capability to reduce VZV infection in 3D human epidermal skin model.

## Discussion

VZV is a widespread human alpha-herpesvirus that cause two distinct diseases, varicella and zoster, respectively. Currently, there is no specific antiviral drugs under clinical. The absence of effective and specific therapies has posed a major threat to public health. In this study, we screened the ideal anti-VZV target based on RNAi mechanism and found that transdermal administration of antiviral r/si-ORF7 could inhibit VZV infection, which suggested that r/si-ORF7 was a promising candidate for functional cure VZV infection. Our study firstly revealed the feasibility to develop anti-VZV siRNA drugs based on RNAi mechanism.

Emerging evidences indicate that RNAi is an important mechanism for resisting viral infection by silencing the specific viral gene. The application of siRNA therapeutics for viral infection is widely used in clinical trials. In this study, we screened the ideal siRNA targets against VZV infection among 21 ORFs according to literature analysis [28] and found ORF7 was critical for VZV replication thus had potential to serve as target of anti-VZV siRNA (Fig. 1). Our result was consistent with the previously study demonstrated that virus with ORF7 deletion mutant (ORF 7D) had a growth defect in skin organ culture (SOC) [28]. Subsequently, ORF7 was identified that have a severe growth defect in dorsal root ganglia (DRG) tissue ex- vivo/in vivo [31]. Moreover, ORF7 affect secondary envelopment and cell-to-cell spread of VZV in differentiated neuronal cells in vitro [32, 33]. Recently, wang et al. revealed ORF7 interacted with ORF53, which played a role in its trans-Golgi network localization during infection [34]. According to the above studies, ORF7 may be an attractive target for developing novel siRNA therapies against VZV infection.

Currently, chemically engineered siRNA reagents have been utilized for plenty of studies. Recently a novel hybrid tRNA^Ser^ scaffold technology to produce highly-structure, stable natural siRNA reagents with minimal posttranscriptional modifications was established (Fig. 2B) [18]. Thus, we used this bio-engineering approach to manufacture recombinant siRNA agents with high yield in *E. coli* and found that r/si-ORF7 could reduce VZV copy numbers significantly, and was selectively processed to mature si-ORF7 in human ARPE-19 cells, which consequently modulated target gene expression (Fig. 2). Further studies verified r/si-ORF7 could inhibit VZV v-Oka and r-Oka replication without affecting cell viability (Fig. 3). The results indicated that bio-engineering recombinant siRNA are biologically active to regulate the target gene expression, in line with the previous findings on the control of target gene expression by recombinant RNA. Furthermore, we blasted the si-ORF7 sequence compared with Standard databases by the National Center of Biotechnology Information website and found si-ORF7 could bind to *Solanum pinnatisectum*, *Solanum pinnatisectum*, and VZV, which indicated that mature si-ORF7 could avoid off-target effects.

Transdermal drug delivery system is a noninvasive method that can avoid the liver first pass effect and prevent gastrointestinal irritation. Topical delivery of siRNAs could be a key to curing skin disease. The application of siRNA therapy for skin disease is highly restricted to the unique barrier properties of human skin, which significantly limits the absorption of siRNAs, and thus hampers the efficient delivery of nucleic acid payloads. Although recent advancements demonstrate the power of non-viral vector, such as lipid-based nanoparticles (LNP), polymeric nanoparticles, and physical delivery method, such as microneedles, for the delivery of the gene therapy materials to the skin, it remains a great challenge due to low delivery efficiency for non-viral vector or small cover surface areas for physical methods [30]. To achieve the transdermal delivery of r/si-ORF7, flexible nano-liposomes was adopted. we prepared and characterized flexible nano-liposomes to delivery r/si-ORF7 (Fig. 4A, B), and confirmed that flexible nano-liposomes could delivery r/si-ORF7 successfully into the cells (Fig. 4 C, D).

VZV is renowned for its very low titer when grown in cultured cells and cell-associated nature because newly formed virions were degraded in late endosomal before exocytosis, producing a large number of noninfectious virions in the supernatant [35–37]. VZV is also remarkably hosting specific which naturally occurring reservoir is in humans, which leads to the absence of suitable animal models and hinders the antiviral drugs development [38, 39]. To overcome this limitation, skin organ culture (SOC) or human tissue xenografts in mice with severe combined immunodeficiency (SCID), have been developed to evaluate VZV infection in different human cells in vivo [39–41]. However, human tissues are expensive, hard to obtain, and are often complicated to generate. To overcome this problem, we established 3D human epidermal skin tissue which was similar to human skin tissue and comprised of four parts: stratum corneum, stratum granulosum, stratum spinosum, and stratum basale (Fig. 5). We then evaluated that the activity of r/si-ORF7 against both VZV v-Oka and r-Oka. As shown in Fig. 6, r/si-ORF7 could inhibit the VZV infection, and protect epidermal skin structure from VZV damage compared with SSA. Hence, r/si-ORF7 had potent capability to reduce VZV infection in 3D human epidermal skin model. The results suggest that r/si-ORF7 could inhibit VZV infection in vitro. VZV infection can lead to abundant fusion and syncytium formation, which was an irreversible process [42]. Hence, it remains a challenge for antivirals to address the cytotoxicity of VZV on the cell line and human organoids after infection.

## Conclusions

Overall, the current findings identify the ORF7 was a promising therapeutic target and found that r/si-ORF7, produced by a novel hybrid tRNA^ser^ scaffold and delivered by flexible nano-liposomes, could inhibit VZV infection effectively in cellular and 3D human epidermal model. These results suggest that r/si-ORF7 can be a potential therapeutic candidate to treat VZV infection uesd in clinic. In the future, the continued development novel model systems are necessary to evaluate the antiviral efficacy of r/si-ORF7.

## Methods

### Cells, antibodies, and reagents

Human retinal pigment epithelial ARPE-19 cells (CL-0026) and Human immortalized keratinocytes HaCaT cells (CL-0090) were purchased from Procell Life Science &Technology (Co., Ltd,), and 0.1% penicilin-Dulbecco’s modified Eagle’s medium (DMEM, Corning) with 10% fetal bovine serum (BI) and 0.1% penicillin-streptomycin-gentamycin solution (Solarbio) at 37 °C with 5% CO_2_. Human primary keratinocytes were purchased from Guangdong (China) Biocell Biotech Co. Ltd., and 0.1% penicilin-KcGrowth medium (PY3011) with 0.1% penicillin-streptomycin-gentamycin solution (Solarbio) at 37 °C with 5% CO_2_. KcGrowth medium (PY3011) and EpiGrowth medium (PY3021) also were purchased from Guangdong (China) Biocell Biotech Co. Ltd. A monoclonal mouse antibody against VZV gE was purchased from Abcam (ab272686). Secondary goat anti-mouse IgG was from Sangon (Shanghai, China). The Monoclonal mouse GAPDH antibody was purchased from Proteintech (Wuhan, China). Infrared dye- labeled anti-mouse antibody was purchased from LI-COR Biosciences (Lincoln, NE, USA). All chemistry siRNA were purchased from GenePharma (Shanghai, China) and the sequence were listed in Additional file 1: Table S1. Cholesterol, 1,2-dioleoyl-3-trimethylammonium-propane chloride salt (DOTAP), 1,2-dioleoyl-sn-glycero-3-phosphoethanolamine (DOPE) were purchased from J&K CHEMICA (Beijing, China).

### Production of recombinant siRNA

Recombinant ORF7-siRNA (r/si-ORF7) and negative control RNA as abbreviated as SSA were conducted as previously described [21, 24, 25]. Briefly, the r/si-ORF7 expression plasmid was constructed by using a novel hybrid tRNASer scaffold (Fig. 1B and Additional file 1: Figure S3) and overexpressed in *E. coli* (HST08). Total RNA of *E.coli* was extracted and purified by fast protein liquid chromatography (FPLC). The purity of r/si-ORF7 was checked by denaturing 8% urea PAGE. The whole manufacture process was performed by RQCON Biological Technology Co., Ltd.(Xi’an, China).

### VZV propagation

VZV Oka vaccine strain (VZV v-Oka) and recombinant Oka (r-Oka) carrying both green fluorescent protein (GFP) and luciferase reporter genes were gifted by Tong Cheng professor (Xiamen University) and Hua Zhu professor (Rutgers University) respectively [28, 43, 44]. ARPE-19 cells were infected with VZV v-Oka or r-Oka at a multiplicity of infection (moi) of 0.3. The viruses were propagated in harvested when the cytopathic effect (CPE) reaching > 80%.VZV-infected ARPE-19 cells were frozen in FBS with 10% DMSO, stored in liquid nitrogen (LN_2_). To better mimic VZV cell to cell transmission character in vitro and in vivo, VZV-infected ARPE-19 cells (CPE > 80%) were selected to infect normal ARPE-19 Cells in the next all experiments.

### Western blot

ARPE-19 cells were seeded in 6-well plate at a density of 2 × 10^5^ cells per well and incubated overnight. The cells were then transfected with 40 nM chemical siRNA or 10 nM r/si-ORF7 with lipofectamine 2000 reagent as described in the manufacture protocol. Complexes were removed after 6 h transfection and cells were infected VZV v-Oka at an moi of 0.3. This moi was chosen to ensure a higher infection level of ARPE-19 cells at harvest timepoint (48 h post infection, 48 hpi). After 48 hpi. Cells were collected and then lysed with RIPA Lysis Buffer and 100×protease inhibitors cocktail (TargetMol, C0001). 30 µg aliquot of each cell lysate was electrophoresed through a 10% polyacrylamide gel and transferred to a 0.22 μm PVDF membrane (Millipore). The membrane was incubated with the primary antibodies against VZV gE and GAPDH, followed by secondary antibodies labeled with infrared dye. The Membrane was visualized using the Odyssey Infrared Imaging System (LI-COR Bioscience).

### Immunofluorescence (IF) assays

As described above transfection and infection protocol. After 48 hpi, cells were washed twice with DPBS and fixed with 4% paraformaldehyde (PFA) for 15 min at room temperature followed by 0.5% Triton X-100 permeabilization for 10 min and 5% BSA blocking for 30 min at room temperature. VZV gE monoclonal mouse antibody and a secondary goat anti-mouse IgG were used to detect the VZV gE protein. 4ʹ,6-Diamidino-2-phenylindole (DAPI, 1 µg/mL) was used to stain the cell nuclei, the plate was observed using a BX60 fluorescence microscope (Olympus, Tokyo, Japan). The intensity of fluorescence was calculated by the ImageJ software.

### RNA isolation and reverse transcription quantitative real-time PCR (RT-qPCR)

As described above transfection and infection protocol. After 48 hpi, total RNA was isolated with TRIzol™ Reagent (Invitrogen) according to the manufacturer’s instructions. Reverse-transcription quantitative real-time PCR system (RT-qPCR) analysis was conducted on a CFX96 Touch Real-time PCR system (Bio-Rad). Quantification of tRNA scaffold, r/si-ORF7 precursor, and mature si-ORF7 mRNA expression level were performed with a 2-step RT-qPCR system (Yesen, China) using gene-selective primers, and stem-loop RT-qPCR analysis of mature si-ORF7 with the 1st strand cDNA synthesis kit (Yesea, China). The thermal cycling protocol was as follows: 95 °C for 2 min followed by 39 cycles consisting of 95 °C for 2 s, 60 °C for 30 s. The relative expression was calculated using the formula 2^−ΔCT^, where was the difference in C_T_ value between the analyte (tRNA scaffold, r/si-ORF7 precursor or mature si-ORF7). GAPDH was used as internal control for the assessment of mRNA level and tRNA scaffold, r/si-ORF7 precursor were normalized to 18 S, and mature si-ORF7 were normalized to U6. Cells were treated in triplicate and assayed separately. The comparative threshold cycle (C_T_) method with formula 2^−ΔCT^ was used to calculate the relative gene expression. All primers were listed in Additional file 1: Table S2.

### Digital PCR (dPCR)

QX200 Droplet Digital PCR (ddPCR) system (Bio-Rad, USA) was used to determine VZV copy numbers decrease triggered by r/si-ORF7. RNA Samples were similarly made as above RT-PCR process. The ddPCR reaction mixture consisted of 10 µL ddPCR 2×Supermix, 0.7 µL forward primer; 0.7 µL reverse primer; 0.6 µL specific probe (10 µM), 1 µL cDNA (0.5 ng/µL), and nuclease-free H_2_O in a final volume of 20 µL. VZV ORF68 -specific primers and probe were purchased by Tsingke Biotechnology Co., Ltd. The entire reaction mixture was placed in the QX200 Droplet Generator (Bio-Rad). After processing, the droplets generated from each sample were transferred to a 96-well PCR plate (Bio-Rad) and heat-sealed with PX1™ PCR Plate Sealer (Bio-Rad) using a thermal profile of beginning: 1 cycle of 95 °C for 1 min, followed by 40 cycles of 95 °C for 15 s and 60 °C for 30 s, and ending at 4 °C. After amplification, the plate was loaded on the QX200 Droplet Reader (Bio-Rad) and the droplets from each well of the plate were read automatically. Positive droplets, containing amplification products, were partitioned from negative droplets by applying a fluorescence amplitude threshold in QuantaSoft™ analysis software (Bio-Rad). Quantification of the target molecules were presented as the number of copies per µL of the PCR mix. The r/si-ORF7 and SSA were tested in three biological replicates. All primers were listed in Additional file 1: Table S3.

### Luciferase assay

ARPE-19 cells were seeded in 12-well plate at a density of 1 × 10^5^ cells per well and incubated overnight. The cells were then transfected with 10 nM r/si-ORF7 and SSA with lipofectamine 2000 reagent according to the manufacturer’s instructions. 6 h later, discarded medium and VZV r-Oka infected normal ARPE-19 (moi = 0.3). From 1 to 4 dpi, the 12-plate were observed using a BX60 fluorescence microscope (Olympus, Tokyo, Japan) and then detected the activity of luciferase. Luciferase signal intensity according to Dual-luciferase® Reporter Assay System (Promega) according to the manufacturer’s instructions with GLOMA (Promega).

### Cell viability assay

The cytotoxicity of r/si-ORF7 on human ARPE-19 cell was determined by CCK8 assay kit (Biosharp, China). Briefly, ARPE-19 cells were seeded in 96-well plate at a density of 1 × 10^4^ cells per well and incubated overnight. The cells were then transfected with 0 nM to 100 nM r/si-ORF7. After 48 h, CCK8 solution was then added to each well and incubated for 4 h. The effect of r/si-ORF7 on cells was measured by absorbance at 450 nm using a spectrophotometer (Synergy HT, Bio-Tek, USA).

The cytotoxicity of flexible nano-liposomes to ARPE19 cells. The ARPE-19 cells were seed in 96-well plate and incubated overnight. 400 ng flexible nano-liposomes (The working concentration of flexible nano-liposomes used in 96-well plate) were added into cells 6 h and then refreshed by 10% DMEM medium. After 48 h, the effect of flexible nano-liposomes on cells was measured as above described.

### Preparation and characterization of flexible nano-liposomes

Flexible nano-liposomes were prepared as previously described. Briefly, moderate DOTAP, DOPE and Cholesterol were dissolved in a mixture solution of with chloroform and methanol. Then, the organic solvents were removed by evaporation for 1 h in 40 °C conditions and the thin-film were vacuum drying for 1 h, and stored in − 20 °C overnight. The obtained thin-film was then hydrated with RNAase free water to get flexible nano-liposomes. In order to obtain uniformly distributed liposomes, the above mixture was extruded through 0.22 μm polycarbonate membranes for 3 times. Polyplexes were prepared using 2 µg of r/si-ORF7 in a total volume of 1 mL of DEPC water. The hydrodynamic diameter and zeta potential of the nanocomplexes was measured using a Nano-ZS analyzer (Nano ZS/ZEN3600, Malvern Panalytical, UK) operating at 25 °C.

Polyplexes were prepared using 2 µg of r/si-ORF7 in a total volume of 1 mL of DEPC water. The hydrodynamic diameter and zeta potential of the nanocomplexes was measured using a Nano-ZS analyzer (Nano ZS/ZEN3600, Malvern Panalytical, UK) operating at 25 °C. The morphologies of the complexes were observed by transmission electron microscopy (TEM, Hitachi HT7800 system, Japan) and images were obtained at an acceleration voltage of 80.0 kV following phosphotungstic acid staining.

### Cellular uptake

The RNA labeled with FAM was used to observe the cellular uptake behavious by using the fluorescence microscope. HaCaT cells were seeded in a 12-well plate at a density of 1 × 10^5^ cells per well and incubated overnight. The ratio weight of liposomes and RNA^FAM^ polyplexes were prepared at 5:1 Stocks of liposomes and RNA^FAM^ were diluted to in Opti-MEM™ medium (Thermo Fisher Scientific) to research the desired concentration. An equal volume of liposome was added to a defined amount of r/si-ORF7 solution and incubated for 20 min. Then the polyplexes were added into cells in each well. Then the cells were stained with a certain concentration of Hoechst for 3 min. Finally, the fluorescence uptake of cells was observed at different time.

### The delivery efficiency of r/si-ORF7 with flexible nano-liposomes

HaCaT cells were seeded in a 12-well plate at a density of 1 × 10^5^ cells per well and incubated overnight. The cells were then transfected with 10 nM r/si-ORF7 with flexible nano-liposomes as described above. After 48 h, total RNA was isolated with TRIzol™ Reagent and the expression level of mature si-ORF7 was detected by stem loop RT-qPCR.

### Preparation 3D human epidermal skin tissue model and r/si-ORF7 activity against VZV

Human primary Keratinocytes were seeded into the culture inserts (BIOFIL, 0.4 μm) with a density of 2 × 10^5^ per tissue, followed by submerged cultured in KcGrowth medium for 24 h. The inserts were then elevated to the air-liquid interface with differentiation medium (EpiGrowth). Following 12 days of air-liquid interface culture, examination of fixed and sectioned artificial skin tissue by H&E and observed by microscope. On the 12th day, the 3D human epidermal skin tissue then transfected with 10 nM r/si-ORF7 and SSA with flexible nano-liposomes. 6 h later, discarded medium and infected VZV v-Oka or r-Oka (moi = 0.3). After 48 hpi, examination of fixed and sectioned artificial skin tissue by H&E or IF and observed by microscope.

### Statistical analysis

The statistical analyses of the data were performed with GraphPad Prism version 7.0 software (GraphPad Software, Inc., La Jolla, CA, United States), and Student’s t-test was used. The data was presented as mean ± standard deviation (SD). *P <* 0.05 was considered statistically significant for all comparisons.

### Supplementary Information

**Additional file 1: Figure** **S1.** The gE expression level in ARPE-19 cells infected with VZV-vOka at moi = 0.3 at 2 d.p.i after treatment with chemically synthesized siRNAs at 6 h post-transfection. **Figure S2.** Representative fluorescent images of VZV gE expression in ARPE-19 cells infected with VZV-vOka at moi = 0.3 at 2 d.p.i after treatment with chemically synthesized siRNAs at 6 h post-transfection. gE was stained green and nuclei were labeled with DAPI (blue), scale bars = 200 µm. **Figure S3.** The secondary structure of bioengineered r/si-ORF7 predicted by CentroidFold (http://www.ncrna.org/centroidfold). **Figure S4. **Representative FPLC traces during the purification of r/si-ORF7. **Figure S5.** The cytotoxicity of flexible nano-liposomes to ARPE19 cells. ARPE-19 cells wereseeded in 96-well plate overnight and then incubated with 400 ng flexible nano-liposomes 6 h (The working concentration of flexible nano-liposomes used in 96-well plate). CCK8 assay was undertaken to measure the cell viability of flexible nano-liposomes on ARPE-19 cells. **Table S1.** Chemically synthesized siRNA sequences. **Table S2.** RT-qPCR primer.**Table S3.** Digital PCR primer.

## Data Availability

The datasets used and/or analyzed during the current study are available from. the corresponding author on reasonable request.

## Abbreviations

CPE: Cytopathic effect
DAPI: 4ʹ,6-Diamidino-2-phenylindole
DEPC: Diethylpyrocarbonate
DMEM: Dulbecco’s modified Eagle’s medium
DOPE: 1,2-Dioleoyl-*sn*-glycero-3-phosphoethanolamine
d.p. i: Days post-infection
ddPCR: Droplet Digital PCR
DOTAP: 1,2-Dioleoyl-3-trimethylammonium-propane chloride salt
DRG: Dorsal root ganglia
EBV: Epstein–Barr virus
*E. coli*: *Escherichia coli*
FPLC: Fast protein liquid chromatography
gE: Glycoprotein E
GFP: Green fluorescent protein
GAPDH: Glyceraldehyde-3-phosphate dehydrogenase
HBV: Hepatitis B virus
HIV: Human immunodeficiency virus
IF: Immunofluorescence
LNP: Lipid-based nanoparticles
mRNAs: Messenger RNAs
moi: Multiplicity of infection
ORF7: Open reading frame 7
PHN: Post-herpetic neuralgia
RNAi: RNA interference
RT-qPCR: Real-time quantitative polymerase chain reaction
r-Oka: Recombinant Oka
ser: Serine
siRNAs: Small interference RNAs
SARS-Cov: Severe-Acute-Respiratory-Syndrome Coronavirus
SARS-Cov-2: Severe-Acute-Respiratory-Syndrome Coronavirus-2
SOC: Skin organ culture
SCID: Severe combined immunodeficiency
tRNA: Transfer RNA
TEM: Transmission electron microscope
VZV: Varicella Zoster Virus
VZV v-Oka: VZV Oka vaccine strain

## References

[1] Arvin AM (1999). Management of varicella-zoster virus infections in children. Adv Exp Med Biol.

[2] John AR, Canaday DH (2017). Herpes zoster in the older adult. Infect Dis Clin North Am.

[3] Gruver C, Guthmiller KB. Postherpetic Neuralgia. 2023 Apr 17. In: StatPearls [Internet]. Treasure Island (FL): StatPearls Publishing; 2023 Jan–. PMID: 29630250.

[4] Mehta A, Michler T, Merkel OM (2021). siRNA therapeutics against respiratory viral infections-what have we learned for potential COVID-19 therapies?. Adv Healthc Mater.

[5] Wu R, Luo KQ (2021). Developing effective siRNAs to reduce the expression of key viral genes of COVID-19. Int J Biol Sci.

[6] van den Berg F, Limani SW, Mnyandu N, Maepa MB, Ely A, Arbuthnot P (2020). Advances with RNAi-based therapy for hepatitis B virus infection. Viruses.

[7] Yin Q, Flemington EK (2006). siRNAs against the Epstein Barr virus latency replication factor, EBNA1, inhibit its function and growth of EBV-dependent tumor cells. Virology.

[8] Wooddell CI, Yuen MF, Chan HL, Gish RG, Locarnini SA, Chavez D (2017). RNAi-based treatment of chronically infected patients and chimpanzees reveals that integrated hepatitis B virus DNA is a source of HBsAg. Sci Transl Med.

[9] Yuen MF, Heo J, Jang JW, Yoon JH, Kweon YO, Park SJ (2021). Safety, tolerability and antiviral activity of the antisense oligonucleotide bepirovirsen in patients with chronic hepatitis B: a phase 2 randomized controlled trial. Nat Med.

[10] Scarborough RJ, Gatignol A (2017). RNA interference therapies for an HIV-1 functional cure. Viruses.

[11] Li BJ, Tang Q, Cheng D, Qin C, Xie FY, Wei Q (2005). Using siRNA in prophylactic and therapeutic regimens against SARS coronavirus in Rhesus macaque. Nat Med.

[12] Kelleher AD, Cortez-Jugo C, Cavalieri F, Qu Y, Glanville AR, Caruso F (2020). RNAi therapeutics: an antiviral strategy for human infections. Curr Opin Pharmacol.

[13] Chang YC, Yang CF, Chen YF, Yang CC, Chou YL, Chou HW (2022). A siRNA targets and inhibits a broad range of SARS-CoV-2 infections including Delta variant. EMBO Mol Med.

[14] Ambike S, Cheng CC, Feuerherd M, Velkov S, Baldassi D, Afridi SQ (2022). Targeting genomic SARS-CoV-2 RNA with siRNAs allows efficient inhibition of viral replication and spread. Nucleic Acids Res.

[15] Ponchon L, Beauvais G, Nonin-Lecomte S, Dardel F (2009). A generic protocol for the expression and purification of recombinant RNA in Escherichia coli using a tRNA scaffold. Nat Protoc.

[16] Huang L, Jin J, Deighan P, Kiner E, McReynolds L, Lieberman J (2013). Efficient and specific gene knockdown by small interfering RNAs produced in bacteria. Nat Biotechnol.

[17] Chen QX, Wang WP, Zeng S, Urayama S, Yu AM (2015). A general approach to high-yield biosynthesis of chimeric RNAs bearing various types of functional small RNAs for broad applications. Nucleic Acids Res.

[18] Li PC, Tu MJ, Ho PY, Batra N, Tran MML, Qiu JX (2021). In vivo fermentation production of humanized noncoding RNAs carrying payload miRNAs for targeted anticancer therapy. Theranostics.

[19] Ho PY, Duan Z, Batra N, Jilek JL, Tu MJ, Qiu JX (2018). Bioengineered noncoding RNAs selectively change cellular miRNome profiles for cancer therapy. J Pharmacol Exp Ther.

[20] Yin C, Tian Y, Yu Y, Yang C, Su P, Zhao Y (2020). Mir-129-5p inhibits bone formation through TCF4. Front Cell Dev Biol.

[21] Yin C, Tian Y, Li D, Yu Y, Jiang S, Hou Y (2022). Long noncoding RNA Lnc-DIF inhibits bone formation by sequestering miR-489-3p. iScience.

[22] Cao T, Lu Y, Wang Q, Qin H, Li H, Guo H (2022). A CGA/EGFR/GATA2 positive feedback circuit confers chemoresistance in gastric cancer. J Clin Invest..

[23] Du W, Liu G, Shi N, Tang D, Ferdek PE, Jakubowska MA (2022). A microRNA checkpoint for ca(2+) signaling and overload in acute pancreatitis. Mol Ther.

[24] Li X, Tian Y, Tu MJ, Ho PY, Batra N, Yu AM (2019). Bioengineered miR-27b-3p and mir-328-3p modulate drug metabolism and disposition via the regulation of target ADME gene expression. Acta Pharm Sin B.

[25] Jilek JL, Tian Y, Yu A-M (2017). Effects of MicroRNA-34a on the pharmacokinetics of cytochrome P450 probe drugs in mice. Drug Metab Dispos.

[26] Tian Y, Zhao Y, Yin C, Tan S, Wang X, Yang C (2022). Polyvinylamine with moderate binding affinity as a highly effective vehicle for RNA delivery. J Control Release.

[27] Abdulbaqi IM, Darwis Y, Khan NA, Assi RA, Khan AA (2016). Ethosomal nanocarriers: the impact of constituents and formulation techniques on ethosomal properties, in vivo studies, and clinical trials. Int J Nanomed.

[28] Zhang Z, Selariu A, Warden C, Huang G, Huang Y, Zaccheus O (2010). Genome-wide mutagenesis reveals that ORF7 is a novel VZV skin-tropic factor. PLoS Pathog.

[29] Mo C, Lee J, Sommer M, Grose C, Arvin AM (2002). The requirement of varicella zoster virus glycoprotein E (gE) for viral replication and effects of glycoprotein I on gE in melanoma cells. Virology.

[30] Zhao YP, Han JF, Zhang FY, Liao TT, Na R, Yuan XF (2022). Flexible nano-liposomes-based transdermal hydrogel for targeted delivery of dexamethasone for rheumatoid arthritis therapy. Drug Deliv.

[31] Selariu A, Cheng T, Tang Q, Silver B, Yang L, Liu C (2012). ORF7 of varicella-zoster virus is a neurotropic factor. J Virol.

[32] Grigoryan S, Kinchington PR, Yang IH, Selariu A, Zhu H, Yee M (2012). Retrograde axonal transport of VZV: kinetic studies in hESC-derived neurons. J Neurovirol.

[33] Jiang HF, Wang W, Jiang X, Zeng WB, Shen ZZ, Song YG (2017). ORF7 of varicella-zoster virus is required for viral cytoplasmic envelopment in differentiated neuronal cells. J Virol.

[34] Wang W, Fu W, Pan D, Cai L, Ye J, Liu J (2017). Varicella-zoster virus ORF7 interacts with ORF53 and plays a role in its trans-golgi network localization. Virol Sin.

[35] Carpenter JE, Hutchinson JA, Jackson W, Grose C (2008). Egress of light particles among filopodia on the surface of varicella-zoster virus-infected cells. J Virol.

[36] Cook ML, Stevens JG (1968). Labile coat: reason for noninfectious cell-free varicella-zoster virus in culture. J Virol.

[37] Gershon AA, Sherman DL, Zhu Z, Gabel CA, Ambron RT, Gershon MD (1994). Intracellular transport of newly synthesized varicella-zoster virus: final envelopment in the trans-golgi network. J Virol.

[38] Myers MG, Connelly BL (1992). Animal models of varicella. J Infect Dis.

[39] Mahalingam R, Gershon A, Gershon M, Cohen JI, Arvin A, Zerboni L (2019). Current in vivo models of varicella-zoster virus neurotropism. Viruses.

[40] Lloyd MG, Smith NA, Tighe M, Travis KL, Liu D, Upadhyaya PK (2020). A Novel Human skin tissue model to study varicella-zoster virus and human cytomegalovirus. J Virol.

[41] Zerboni L, Sen N, Oliver SL, Arvin AM (2014). Molecular mechanisms of varicella zoster virus pathogenesis. Nat Rev Microbiol.

[42] Maresova L, Pasieka TJ, Grose C (2001). Varicella-zoster Virus gB and gE coexpression, but not gB or gE alone, leads to abundant fusion and syncytium formation equivalent to those from gH and gL coexpression. J Virol.

[43] Wang W, Pan D, Fu W, Ye X, Han J, Yang L (2022). Development of a skin- and neuro-attenuated live vaccine for varicella. Nat Commun.

[44] Wang W, Zheng Q, Pan D, Yu H, Fu W, Liu J (2020). Near-atomic cryo-electron microscopy structures of varicella-zoster virus capsids. Nat Microbiol.

