# Supplementary material for: A novel recombinant ORF7-siRNA delivered by flexible nano-liposomes inhibits varicella zoster virus infection
Source: Cell Biosci. 2023 Sep 12;13:167. doi: 10.1186/s13578-023-01108-1 (PMC10496174; doi:10.1186/s13578-023-01108-1)
Supplement: Supplementary file 1 — Additional file 1: Figure S1. The gE expression level in ARPE-19 cells infected with VZV-vOka at moi = 0.3 at 2 d.p.i after treatment with chemically synthesized siRNAs at 6 h post-transfection. Figure S2. Representative fluorescent images of VZV gE expression in ARPE-19 cells infected with VZV-vOka at moi = 0.3 at 2 d.p.i after treatment with chemically synthesized siRNAs at 6 h post-transfection. gE was stained green and nuclei were labeled with DAPI (blue), scale bars = 200 µm. Figure S3. The secondary structure of bioengineered r/si-ORF7 predicted by CentroidFold (http://www.ncrna.org/centroidfold). Figure S4. Representative FPLC traces during the purification of r/si-ORF7. Figure S5. The cytotoxicity of flexible nano-liposomes to ARPE19 cells. ARPE-19 cells wereseeded in 96-well plate overnight and then incubated with 400 ng flexible nano-liposomes 6 h (The working concentration of flexible nano-liposomes used in 96-well plate). CCK8 assay was undertaken to measure the cell viability of flexible nano-liposomes on ARPE-19 cells. Table S1. Chemically synthesized siRNA sequences. Table S2. RT-qPCR primer.Table S3. Digital PCR primer. [file 13578_2023_1108_MOESM1_ESM.docx]

**Additional Information**

**
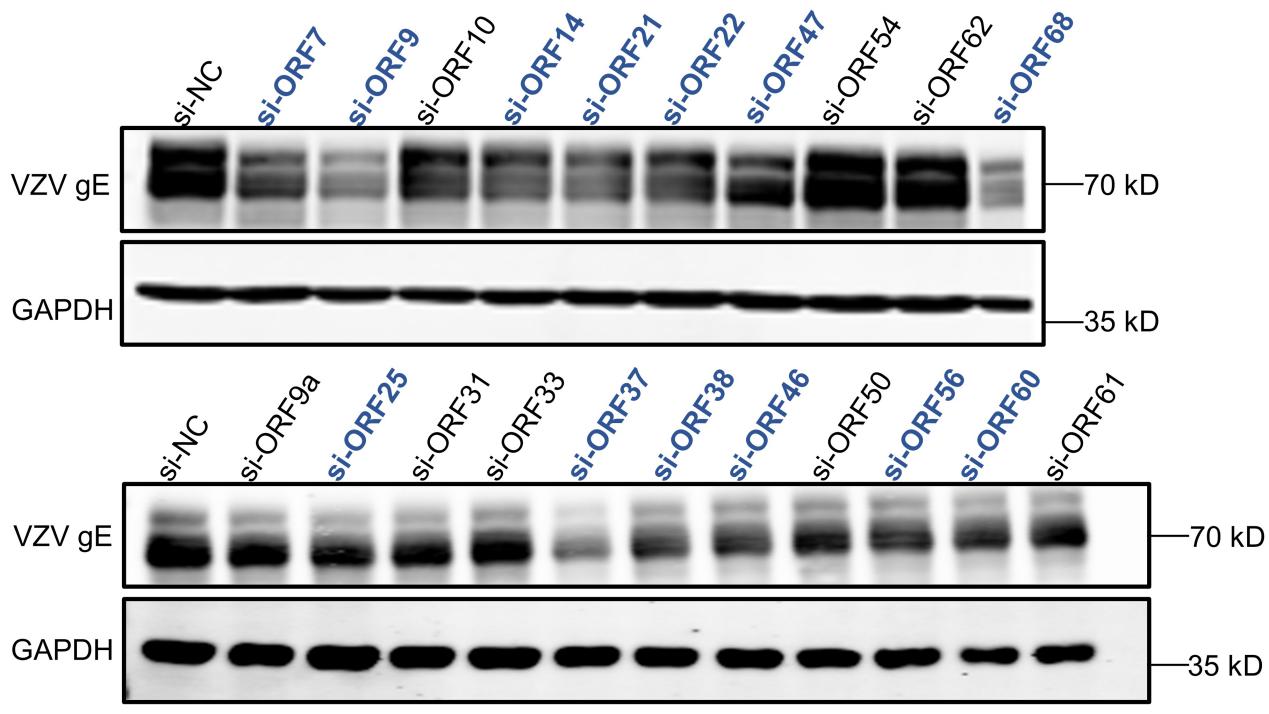
**

**Figure** **S1** The gE expression level in ARPE-19 cells infected with VZV-vOka at moi=0.3 at 2 d.p.i after treatment with chemically synthesized siRNAs at 6 h post-transfection.

**
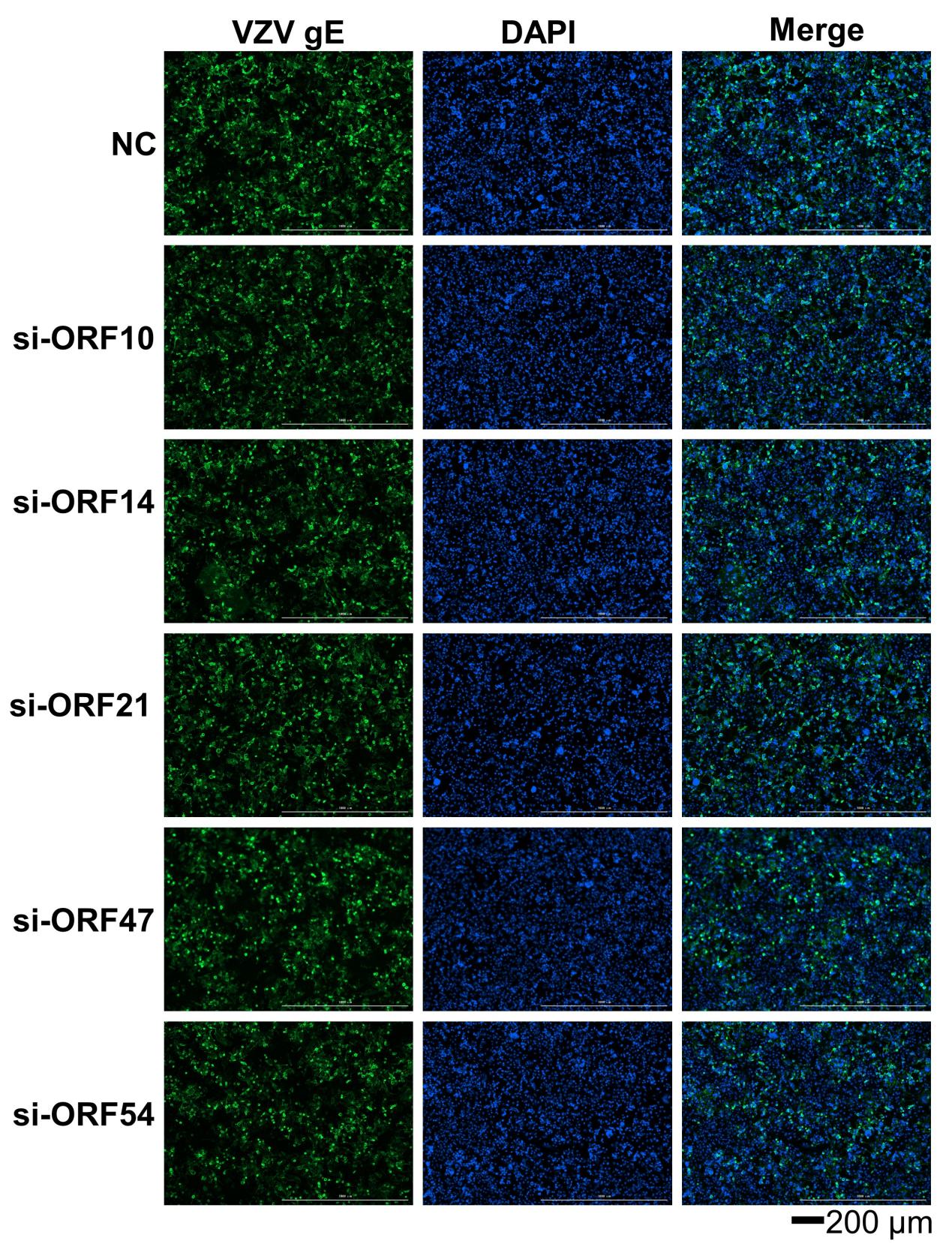
**

**Figure S2** Representative fluorescent images of VZV gE expression in ARPE-19 cells infected with VZV-vOka at moi=0.3 at 2 d.p.i after treatment with chemically synthesized siRNAs at 6 h post-transfection. gE was stained green and nuclei were labeled with DAPI (blue), scale bars = 200 µm.


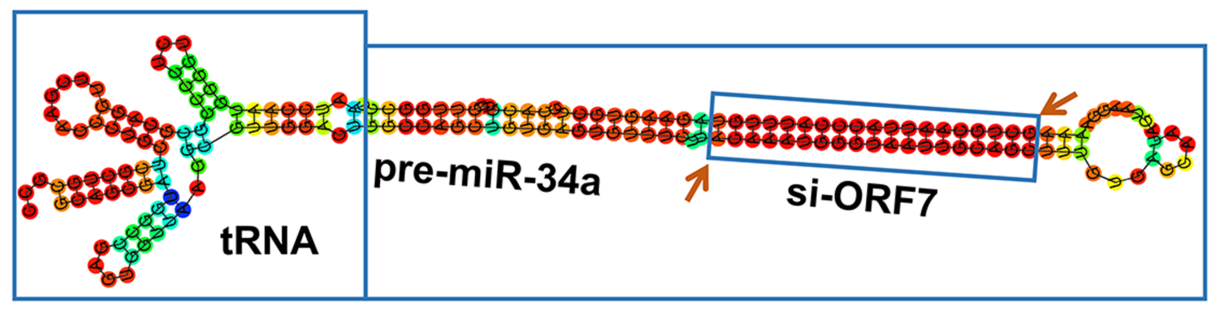


**Figure S3.** The secondary structure of bioengineered r/si-ORF7 predicted by CentroidFold (<http://www.ncrna.org/centroidfold>)

**
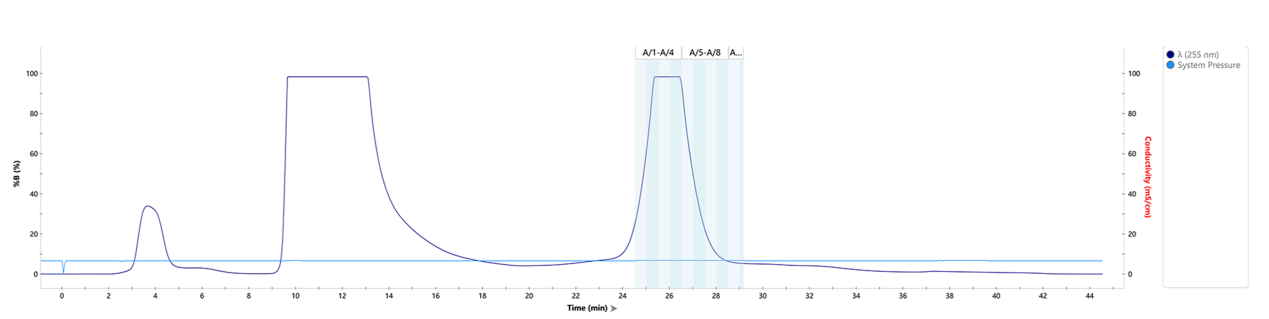
**

**Figure S4.** Representative FPLC traces during the purification of r/si-ORF7


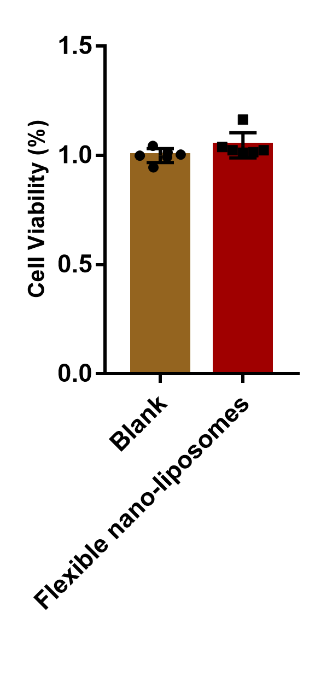


**Figure S5.** The cytotoxicity of flexible nano-liposomes to ARPE19 cells. ARPE-19 cells wereseeded in 96-well plate overnight and then incubated with 400 ng flexible nano-liposomes 6 h (The working concentration of flexible nano-liposomes used in 96-well plate). CCK8 assay was undertaken to measure the cell viability of flexible nano-liposomes on ARPE-19 cells.

Table S1. Chemically synthesized siRNA sequences

| siRNAs | Sequence (5'-3') |
| --- | --- |
| Si-ORF7 | GCUGCAAUUACCCAUUUGUTT (sense)  ACAAAUGGGUAAUUGCAGCTT (antisense) |
| Si-ORF9a | CCGCUUCGUUCAUAUUAAUTT (sense)  AUUAAUAUGAACGAAGCGGTT (antisense) |
| Si-ORF9 | GGGUUACAUUACCACAGUUTT (sense)  AACUGUGGUAAUGUAACCCTT (antisense) |
| Si-ORF10 | GACGACAAUAGGUCUUAAUTT (sense)  AUUAAGACCUAUUGUCGUCTT (antisense) |
| Si-ORF14 | GCCGAAACAUAACUAAAUATT (sense)  UAUUUAGUUAUGUUUCGGCTT (antisense) |
| Si-ORF21 | GCCUUAAAGGAUGCAACAATT (sense)  UUGUUGCAUCCUUUAAGGCTT (antisense) |
| Si-ORF22 | GCAUAUGACAUAUGCGCAUTT (sense)  AUGCGCAUAUGUCAUAUGCTT (antisense) |
| Si-ORF25 | GGCGACUGGUAAAUGAUAUTT (sense)  AUAUCAUUUACCAGUCGCCTT (antisense) |
| Si-ORF31 | GCCCAGGAAAUGAUUAAAUTT (sense)  AUUUAAUCAUUUCCUGGGCTT (antisense) |
| Si-ORF33 | GGUGGAAUGUGGCGUUUAUTT (sense)  AUAAACGCCACAUUCCACCTT (antisense) |
| Si-ORF37 | GGGCGAUUAUGGAUAUAAUTT (sense)  AUUAUAUCCAUAAUCGCCCTT (antisense) |
| Si-ORF38 | GCGCCUAAAUAUGCUAUAUTT (sense)  AUAUAGCAUAUUUAGGCGCTT (antisense) |
| Si-ORF46 | CCUGCGCCGAUUUAAAUAATT (sense)  UUAUUUAAAUCGGCGCAGGTT (antisense) |
| Si-ORF47 | GGGCCUUACUAAAUAUACUTT (sense)  AGUAUAUUUAGUAAGGCCCTT (antisense) |
| Si-ORF50 | GCCGUGAUCAGUGUAUAUATT (sense)  UAUAUACACUGAUCACGGCTT (antisense) |
| Si-ORF54 | GCUUUGAGUUACCAAGAAUTT (sense)  AUUCUUGGUAACUCAAAGCTT (antisense) |
| Si-ORF56 | GACGUUCUUUCGUUAAUAUTT (sense)  AUAUUAACGAAAGAACGUCTT (antisense) |
| Si-ORF60 | GCCAUUUGUUUGUCUUUAATT (sense)  UUAAAGACAAACAAAUGGCTT (antisense) |
| Si-ORF62 | GGAGUUCGUAAACGAUCAUTT (sense)  AUGAUCGUUUACGAACUCCTT (antisense) |
| Si-ORF68 | GGACCAACCGUGGAUUGUUTT (sense)  AACAAUCCACGGUUGGUCCTT (antisense) |
| Si-ORF61 | GCCCGUAAAUACCUAUAUATT (sense)  UAUAUAGGUAUUUACGGGCTT (antisense) |

Table S2. RT-qPCR primer

| Gene | Sequence (5'-3') |
| --- | --- |
| GAPDH | Forword: TGCACCACCAACTGCTTAG  Reverse: GGATGCAGGGATGATGTTC |
| U6 | Forword: CCTCTGACTTCAACAGCGAC  Reverse: TCCTCTTGTGCTCTTGCTGG |
| 18s | Forword: GTAACCCGTTGAACCCCATT  Reverse: CCATCCAATCGGTAGTAGCG |
| tRNA scaffold | Forword: ACGTAGCTCAGTTGGTTAGAGCAG  Reverse: GTGGCTACGACGGGATTCGA |
| Precursor of mature si-ORF7 | Forword: CCGGGCCAGCTGTGAGTGTTTC  Reverse: GGGCCAACAACGTGCAGC |
| ORF7 Stem loop RT- primer | GTCGTATCCAGTGCAGGGTCCGAGGTATTCGCACTGGATACGACAAGCTG |
| Mature si-ORF7 | Forword: GCGCGACAAATGGGTAATTG  Reverse: AGTGCAGGGTCCGAGGTATT |
| ORF7 | Forword: TGGGGTCGTTGCTAAACCTC  Reverse: TCTTGCGTCTGTTTTGGGGT |

Table S3. Digital PCR primer

| Gene | Sequence (5'-3') |
| --- | --- |
| ORF68-F | GCACCGATTCAGCGGATTT |
| ORF68-R | CGGCAAAAAGCTCCAAGTCT |
| ORF68-probe | (FAM) TGGAGTCCGGTACACC (MGB) |

**Raw image files**

| **Fig. 3B** | |
| --- | --- |
| 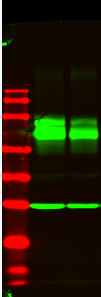 | 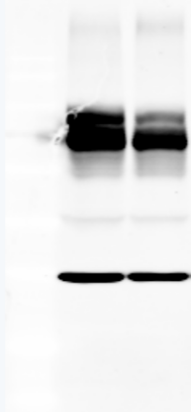 |

**Fig S1：**

| Upper | Low exposure (Lower) | High exposure (Lower) |
| --- | --- | --- |
| 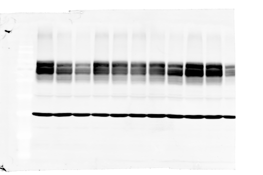 | 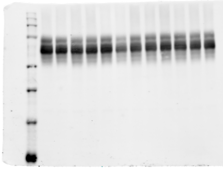 | 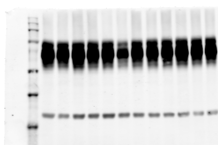 |

The Membrane was visualized using the Odyssey Infrared Imaging System (LI-COR Bioscience)
